# Gut microbiota derived metabolite trimethylamine N-oxide influences prostate cancer progression via the p38/HMOX1 pathway

**DOI:** 10.3389/fphar.2024.1526051

**Published:** 2025-01-09

**Authors:** Yuhua Zhou, Jing Lv, Shengkai Jin, Chaowei Fu, Bo Liu, Yang Shen, Menglu Li, Yuwei Zhang, Ninghan Feng

**Affiliations:** ^1^ Wuxi School of Medicine, Jiangnan University, Wuxi, China; ^2^ Medical School of Nantong University, Nantong, China; ^3^ Jiangnan Medical Center, Nanjing Medical University, Nanjing, China; ^4^ Department of Urology, Jiangnan University Medical Center, Wuxi, China

**Keywords:** trimethylamine N-oxide, gut microbiota, prostate cancer, HMOX1, high-choline diet

## Abstract

**Background:**

Prostate cancer was the fourth most diagnosed cancer worldwide in 2022. Radical treatments and androgen deprivation therapy benefit newly diagnosed patients but impact quality of life, often leading to castration-resistant prostate cancer. Short-term dietary changes significantly affect the gut microbiota, which differs markedly between prostate cancer patients and healthy individuals, impacting both cancer progression and treatment response. A high-choline diet increases the risk of fatal prostate cancer, potentially mediated by the conversion of choline to the trimethylamine N-oxide (TMAO) by the gut microbiota.

**Methods:**

The CCK8 assay was employed to investigate whether TMAO affects the proliferation ability of prostate cancer cells and to determine the appropriate drug concentration. Subsequently, CCK8 time gradients, colony formation assays, and EdU assays measured TMAO’s influence on cell proliferation. Wound healing and transwell migration assays evaluated TMAO’s effect on cell migration. RNA-seq analysis was performed to explore the mechanisms by which TMAO influences the proliferation and migration of prostate cancer cells. qPCR and Western blotting were utilized to validate the expression of related mRNA or proteins. Finally, we performed *in vivo* experiments to evaluate the effect of a high choline diet on the growth of subcutaneous tumors and lung metastases in mice.

**Results:**

Our study found that TMAO enhances the proliferation and migration of prostate cancer cells by upregulating HMOX1 via activation of the MAPK signaling pathway, specifically p38 MAPK. In mouse subcutaneous tumor and lung metastatic tumor experiments, the high-choline diet increased prostate cancer cell proliferation and migration, resulting in significantly greater tumor volume and number of metastases than controls.

**Conclusion:**

This study is the first to demonstrate the role of the gut microbiota-derived metabolite TMAO in prostate cancer. TMAO promotes the proliferation and migration of prostate cancer cells by activating the p38 pathway and increasing HMOX1 expression. Reducing choline intake through dietary intervention may delay the onset and progression of prostate cancer, presenting significant clinical application value.

## Introduction

Prostate cancer (PCa) was the fourth most diagnosed cancer globally in 2022 and the second most common cancer among men (Bray et al., 2024). For patients with early-stage prostate cancer, although radical treatment or androgen deprivation therapy can provide potential therapeutic benefits, these treatments often significantly reduce the quality of life. Additionally, despite postoperative management through monitoring blood levels of prostate-specific antigen, a significant number of patients inevitably progress to castration-resistant prostate cancer (CRPC) (Sharifi et al., 2005). In recent years, although new treatment methods such as targeted therapy, endocrine therapy, and immunotherapy have improved patient survival rates, the overall prognosis remains poor (Sallam et al., 2024; Roach, 2016; Pulido et al., 2024). Therefore, exploring the mechanisms that promote PCa progression to delay or improve patient prognosis is of utmost importance.

The gut microbiota, referred to as the body’s “second genome,” plays a crucial role in various physiological, pathological, and metabolic processes (Turnbaugh et al., 2007). Studies have shown that the composition of the gut microbiome differs between men with and without prostate cancer (Liss et al., 2018). Recent research has further confirmed that the gut microbiota can influence the progression of prostate cancer (Pernigoni et al., 2023). Pernigoni et al. reported that in patients receiving AR-targeted therapy, the gut microbiota can catalyze the synthesis of androgenic steroids, leading to disease progression to CRPC (Pernigoni et al., 2021). Another study on the gut microbiota and immunotherapy indicated that an increase in *Streptococcus salivarius* and a decrease in *Akkermansia muciniphila* in feces were associated with a positive response to anti-PD1 immunotherapy (Peiffer et al., 2022). Overall, compared to healthy individuals, the gut microbiota of prostate cancer patients undergoes significant changes, and these changes, depending on the specific composition of the microbiota, affect the patient’s prognosis and response to immunotherapy and other treatments.

Short-term dietary changes have been shown to significantly affect the gut microbiota (David et al., 2014; Jumpertz et al., 2011). A high-fat diet is considered a major risk factor for prostate cancer, with studies indicating that it promotes prostate cancer growth through histamine signaling by mast cells (Matsushita et al., 2022). Additionally, high choline diets have been found to increase the risk of lethal prostate cancer, although the specific mechanisms remain unclear (Richman et al., 2012). Previous research has demonstrated that choline is metabolized by the gut microbiota into trimethylamine (TMA), which is subsequently oxidized in the liver to form trimethylamine-N-oxide (TMAO) (Wang et al., 2011). Extensive studies have confirmed that a high-choline diet significantly increases plasma TMAO levels (Romano et al., 2015; He et al., 2024). Therefore, we hypothesize that TMAO produced from a high-choline diet may influence the progression of prostate cancer.

TMAO is a gut microbiota-derived metabolite whose substrates include phosphatidylcholine, choline, betaine, and L-carnitine, which are enriched in seafood, dairy products, eggs, red meat, and organ meats (Cho and Caudill, 2017). These nutrients are metabolized into TMA in the gut, absorbed into the bloodstream, and oxidized to TMAO in the liver by flavin-containing monooxygenase 3 (Wang et al., 2011). Previous studies have reported that TMAO is associated with various health outcomes, including all-cause mortality, cardiovascular disease, hypertension, diabetes, and chronic kidney disease (Li et al., 2022). TMAO can activate inflammatory pathways, induce reactive oxygen species (ROS) production, impair glucose tolerance, and hinder insulin signaling (Seldin et al., 2016; Chen et al., 2017; Gao et al., 2014). Recently, attention has also turned to the impact of TMAO in cancer. TMAO activates the NLRP3 inflammasome, inducing colorectal cancer cell proliferation and promoting angiogenesis through high levels of vascular endothelial growth factor A (Yue et al., 2017; Yang et al., 2022). Moreover, TMAO promotes ROS production, leading to increased proliferation of normal liver parenchymal cells and a significant increase in hepatocellular adenoma incidence (Shen et al., 2003). Mechanistically, Wu et al. revealed that TMAO promotes hepatocellular carcinoma cell proliferation, migration, and invasion through the POSTN/ILK/AKT/mTOR axis (Wu et al., 2022). Interestingly, TMAO has also been reported to enhance the anti-PD1 immunotherapy response in triple-negative breast cancer and pancreatic cancer, suggesting a complex role for TMAO in different cancer contexts (Wang et al., 2022; Mirji et al., 2022). Given its significant associations with various diseases, TMAO is considered a novel gut microbiota-related biomarker representing human health status, warranting further investigation into its role in other diseases (Li et al., 2022).

Here, our study is the first to demonstrate that the gut microbiota-derived metabolite TMAO can promote the malignant progression of prostate cancer cells. Through transcriptomic analysis, we explored the specific mechanisms involved. Our findings reveal the potential impact of TMAO on prostate cancer, providing theoretical support for the relationship between the gut microbiota and prostate cancer. This is the first study to explain the mechanism of the effect of a high-choline diet on prostate cancer, highlight the importance of dietary habits in prostate cancer prevention, and provide a theoretical basis for future prevention and treatment strategies.

## Materials and methods

### Materials

Normal prostate epithelial cell line RWPE-1 and prostate cancer cell lines LNCaP, DU145, and PC3 were obtained from the American Type Culture Collection (ATCC, United States). Fetal bovine serum (FBS, Cat. CTCC-002-071-50) was purchased from Meisen CTCC (Zhejiang, China). K-SFM and EMEM cell culture media were obtained from Gibco (Shanghai, China). RPMI-1640 cell culture medium was purchased from Cytiva (Shanghai, China), and F12k cell culture medium was obtained from Boster (Wuhan, China). Penicillin-Streptomycin was purchased from Gibco (Cat. 15140122, Shanghai, China). TMAO was purchased from Sigma-Aldrich (CAS: 1184-78-7, Shanghai, China). BeyoClick™ EdU Cell Proliferation Kit with Alexa Fluor 488 was obtained from Beyotime Biotechnology (Shanghai, China). Hoechst 33258 was purchased from Songon Biotech (Shanghai, China). Cell Counting Kit-8 was obtained from DOJINDO (Shanghai, China). Phosphatase inhibitor cocktails were purchased from Abcam (ab201113), and Protease Inhibitor Cocktail (Cat. HY-K0010) was purchased from MedChemExpress (Shanghai, China). Anti-Heme Oxygenase 1 antibody (ab189491) was obtained from Abcam (Shanghai, China). JNK Monoclonal antibody (Cat. 66210-1-lg), p38 MAPK Polyclonal antibody (Cat. 14064-1-AP), ERK1/2 Polyclonal antibody (Cat. 11257-1-AP), Phospho-JNK (Tyr185) Recombinant (Cat. 80024-1-RR), Phospho-p38 MAPK (Thr180/Tyr182) Polyclonal antibody (Cat. 28796-1-AP), Phospho-ERK1/2 (Thr202/Tyr204) Polyclonal antibody (Cat. 28733-1-AP), Beta Actin Monoclonal antibody (Cat. 66009-1-Ig) and Ki-67 Polyclonal antibody (Cat. 27309-1-AP) were all purchased from Proteintech (Wuhan, China). SP600125 (Cat. HY-12041) and SB203580 (Cat. HY-10256) were purchased from MedChemExpress (Shanghai, China). AZD6244 (Cat. T6218) was purchased from Targetmol (Shanghai, China).

### Cell culture and transfection

RWPE-1 cells were cultured in K-SFM medium supplemented with 10% FBS, 100 U/mL penicillin, and 100 U/mL streptomycin. LNCaP cells were cultured in RPMI-1640 medium supplemented with 10% FBS, 100 U/mL penicillin, and 100 U/mL streptomycin. PC3 cells were cultured in F12k medium supplemented with 10% FBS, 100 U/mL penicillin, and 100 U/mL streptomycin. DU145 cells were cultured in EMEM medium supplemented with 10% FBS, 100 U/mL penicillin, and 100 U/mL streptomycin. All cells were maintained at 37°C in a humidified incubator with 5% CO2.

The *HMOX1*-targeting siRNA was purchased from Tsingke Biotech (Tsingke Biotech Co., Ltd., Beijing, China). According to the manufacturer’s instructions, *HMOX1*-siRNA was transfected into DU145 and PC3 cells using Lipofectamine 3000 (Thermo Fisher Scientific). The transfection efficiency was assessed using Western blotting and qPCR. The sequences of *HMOX1*-siRNA are provided in Supplementary Table 1.

### CCK8 assay

To assess the effect of TMAO on the proliferation of RWPE-1, LNCaP, DU145, and PC3 cells, a concentration gradient of TMAO was tested using the CCK8 assay. Cells were seeded in 96-well plates at 2000 cells per well and treated with various concentrations of TMAO for 48 h. After removing the medium, 10 μL of CCK8 reagent was added to each well according to the manufacturer’s instructions and incubated at 37°C for 1 h. Absorbance at 450 nm was measured. For the CCK8 time gradient assay, cells were treated with a fixed concentration of TMAO for up to 96 h, and absorbance was measured every 24 h. Each experiment was repeated three times with n = 6.

### Colony formation assay

DU145 and PC3 cells were seeded in 6-well plates at 1,000 cells per well and treated with DMSO or TMAO. Cells were cultured for approximately 1 week, then fixed with 4% paraformaldehyde for 30 min and stained with 0.1% crystal violet for 25 min. Excess dye was removed by washing with PBS, and colonies were photographed and counted using ImageJ software. Each experiment was repeated three times with n = 3.

### EdU staining

DU145 and PC3 cells were seeded in 6-well plates and treated with TMAO for 48 h. EdU was diluted in cell culture medium at a ratio of 1:1,000 to a final concentration of 10 μM and incubated with cells for 2 h at 37°C. Cells were fixed with 4% paraformaldehyde for 15 min, washed with PBS, permeabilized with 0.3% Triton X-100 in PBS for 15 min, and stained using the Click reaction kit as per the manufacturer’s instructions. Hoechst 33258 was used to stain nuclei for 15 min. Fluorescent images were captured using a fluorescence microscope. Each experiment was repeated three times with n = 3.

### Wound healing assay

DU145 and PC3 cells were seeded in 6-well plates and treated with TMAO for 72 h to form a confluent monolayer. A wound was created using a sterile pipette tip, and cells were washed with PBS. Cells were then cultured in serum-free medium for 24 or 72 h, and images of the wound healing process were captured at each time point. Each experiment was repeated three times with n = 3.

### Transwell migration assay

DU145 and PC3 cells were treated with TMAO for 48 h. Cells were trypsinized, counted, and 2 × 10^4 cells were resuspended in 200 μL serum-free medium and added to the upper chamber of a transwell insert. The lower chamber contained 500 μL medium with 10% FBS. After 24 h of incubation at 37°C, cells were fixed with 4% paraformaldehyde for 30 min and stained with 0.1% crystal violet for 25 min. The upper surface of the transwell insert was wiped clean, and migrated cells on the lower surface were photographed and counted under a microscope. Each experiment was repeated three times with n = 3.

### RNA sequencing

In this study, we performed transcriptome sequencing on six samples, including three control group samples treated with DMSO and three treated group samples, where PC3 cells were treated with 6 mM TMAO. Samples were collected after 48 h of TMAO treatment and immediately stored in liquid nitrogen to maintain RNA integrity and stability. Samples were thawed and ground prior to sequencing, and total RNA extraction was performed according to the manufacturer’s protocol. After quality control, cDNA libraries were constructed and sequenced. Quality-controlled clean data were aligned to the reference genome and counted using featureCounts to obtain the expression of each gene. DESeq2 was used to identify genes with significant differential changes.

### RNA extraction and qRT-PCR

DU145 and PC3 cells were treated with TMAO for 48 h. Total RNA was extracted using the FastPure^®^ Cell/Tissue Total RNA Isolation Kit V2 (Vazyme Biotech Co., Ltd., Nanjing, China). cDNA was synthesized using the HiScript III RT SuperMix for qPCR (Vazyme Biotech Co., Ltd., Nanjing, China). qRT-PCR was performed using ChamQ Universal SYBR qPCR Master Mix (Vazyme Biotech Co., Ltd., Nanjing, China) and an Applied Biosystems QuantStudio 5 system (Thermo Fisher Scientific, United States). Primer sequences for relevant genes are provided in Supplementary Table 1. Each experiment was repeated three times with n = 3.

### Western blotting assay

DU145 and PC3 cells were seeded and treated as described for other experiments. Cells were lysed in RIPA buffer (Beyotime Biotechnology, China) containing Protease Inhibitor Cocktail or Phosphatase Inhibitor Cocktails. Protein concentration was measured using a BCA Protein Assay Kit (Beyotime Biotechnology, China). Equal amounts of protein (30 μg) were separated by 10% SDS-PAGE and transferred onto PVDF membranes. Membranes were blocked with Western Blocking Buffer (Keygen BioTECH, China) for 2 h at room temperature and incubated with primary antibodies overnight at 4°C. After washing with TBST, membranes were incubated with HRP-conjugated secondary antibodies for 1 h at room temperature. Protein bands were visualized using an Enhanced Chemiluminescence Kit (ECL, Keygen BioTECH, China).

### *In vivo* tumor xenograft assay

This study was approved by the Experimental Animal Management and Animal Welfare Ethics Committee of Jiangnan University, with the ethics number JN. No20230830b0361025. Twenty male BALB/c nude mice were purchased from SiPeiFu (Beijing, China) and randomly divided into two groups: a normal diet group (0.14% choline) and a high-choline diet group (1.4% choline). The mice were pre-fed their respective diets for 7 days. PBS and matrigel were mixed at a 1:1 ratio, and 2 × 10^7 PC3 cells were resuspended in 100 μL of the mixture. A 100 μL aliquot of the cell suspension was injected subcutaneously into the axillary region of each mouse. Tumor volume was measured every 7 days for 35 days. Additionally, 1 × 10^7 PC3 cells were resuspended in 100 µL PBS. The cell suspension was injected into mice through the tail vein to establish lung metastasis models. Mice were euthanized using carbon dioxide asphyxiation, and tumors and lungs were excised, weighed, and subjected to further analysis, including HE staining and immunohistochemical staining.

### Immunohistochemistry and histology

Tumor samples were collected and fixed in paraformaldehyde overnight. Samples were washed with PBS, dehydrated with ethanol, embedded, sectioned, and stained. Primary antibodies used for immunohistochemistry were: HMOX1 (1:20,000, Abcam) and Ki-67 (1:2,000, Proteintech). Sections were also stained with hematoxylin and eosin (HE).

### Statistical analysis

All statistical analyses were performed using GraphPad Prism 9.0 (GraphPad Software, Inc., San Diego, CA, United States). Data were presented as mean ± standard deviation. Significant differences were determined using paired or nonpaired two-tailed t-test. Each experiment was repeated three times. A p-value < 0.05 was considered statistically significant. * means p value < 0.05; ** means p value < 0.01; *** means p value < 0.001; **** means p value < 0.0001.

## Results

### TMAO promotes proliferation and migration of prostate cancer cells

**FIGURE 1:**
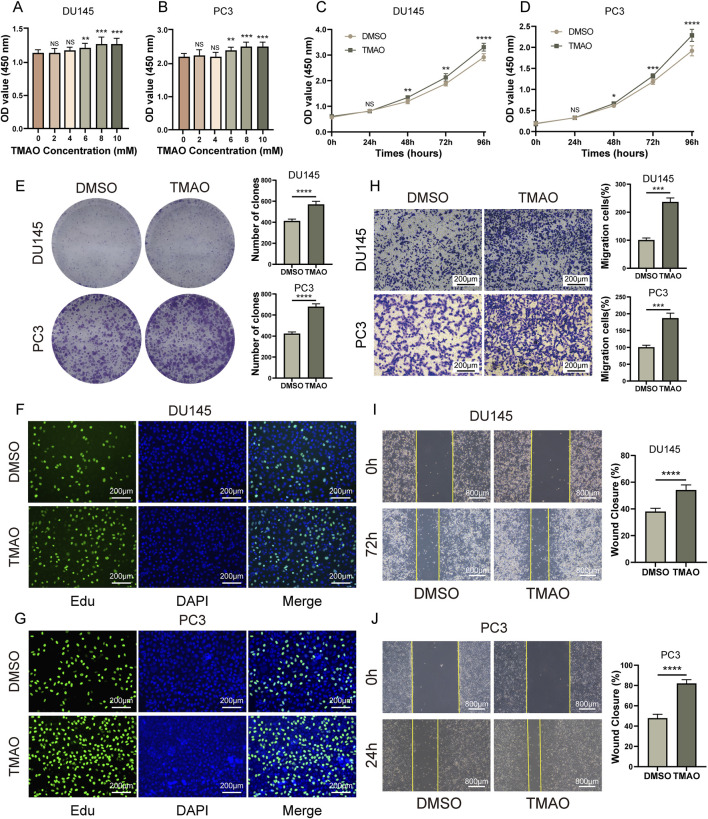
TMAO promotes proliferation and migration of prostate cancer cells. **(A, B)** CCK8 concentration gradient assays in DU145 and PC3 prostate cancer cells to determine TMAO concentrations for subsequent experiments; **(C, D)** CCK8 time gradient assays in DU145 and PC3 cells treated with TMAO; **(E)** Colony formation assays in DU145 and PC3 cells treated with TMAO; **(F, G)** EdU assays in DU145 and PC3 cells treated with TMAO; **(H)** Transwell migration assays in DU145 and PC3 cells treated with TMAO; **(I, J)** Wound healing assays in DU145 and PC3 cells treated with TMAO.

Rapid proliferation of tumor cells is one of the malignant characteristics of cancer. Therefore, we investigated whether TMAO affects the proliferation of prostate cancer cells. In the CCK8 assay, we treated DU145, PC3, RWPE, and LNCaP cells with a gradient of TMAO concentrations (0, 2, 4, 6, 8, 10 mM). We found that at a concentration of 6 mM, TMAO significantly promoted the proliferation of DU145 and PC3 cells (Figures 1A, B), while it had no effect on RWPE-1 and LNCaP cell lines (Supplementary Figures S1A, B). For subsequent experiments, we used a TMAO concentration of 6 mM. In the CCK8 time gradient assay, we treated DU145 and PC3 cells with 6 mM TMAO for 96 h and found a significant difference starting at 48 h (Figures 1C, D). Additionally, the colony formation assay confirmed that TMAO promotes the proliferation of prostate cancer cells (Figure 1E). The Edu assay also showed that more cells were in the proliferation phase after TMAO treatment compared to the control group (Figures 1F, G).

The migration ability of tumor cells is crucial for the malignancy and prognosis of cancer. We used transwell and wound healing assays to investigate whether TMAO affects the migration of tumor cells. Transwell assay results showed that TMAO significantly promoted the migration ability of prostate cancer cells (Figure 1H). The wound healing assay also supported this conclusion (Figures 1I, J). In summary, TMAO promotes the proliferation and migration of prostate cancer cells.

### TMAO increases HMOX1 expression

**FIGURE 2:**
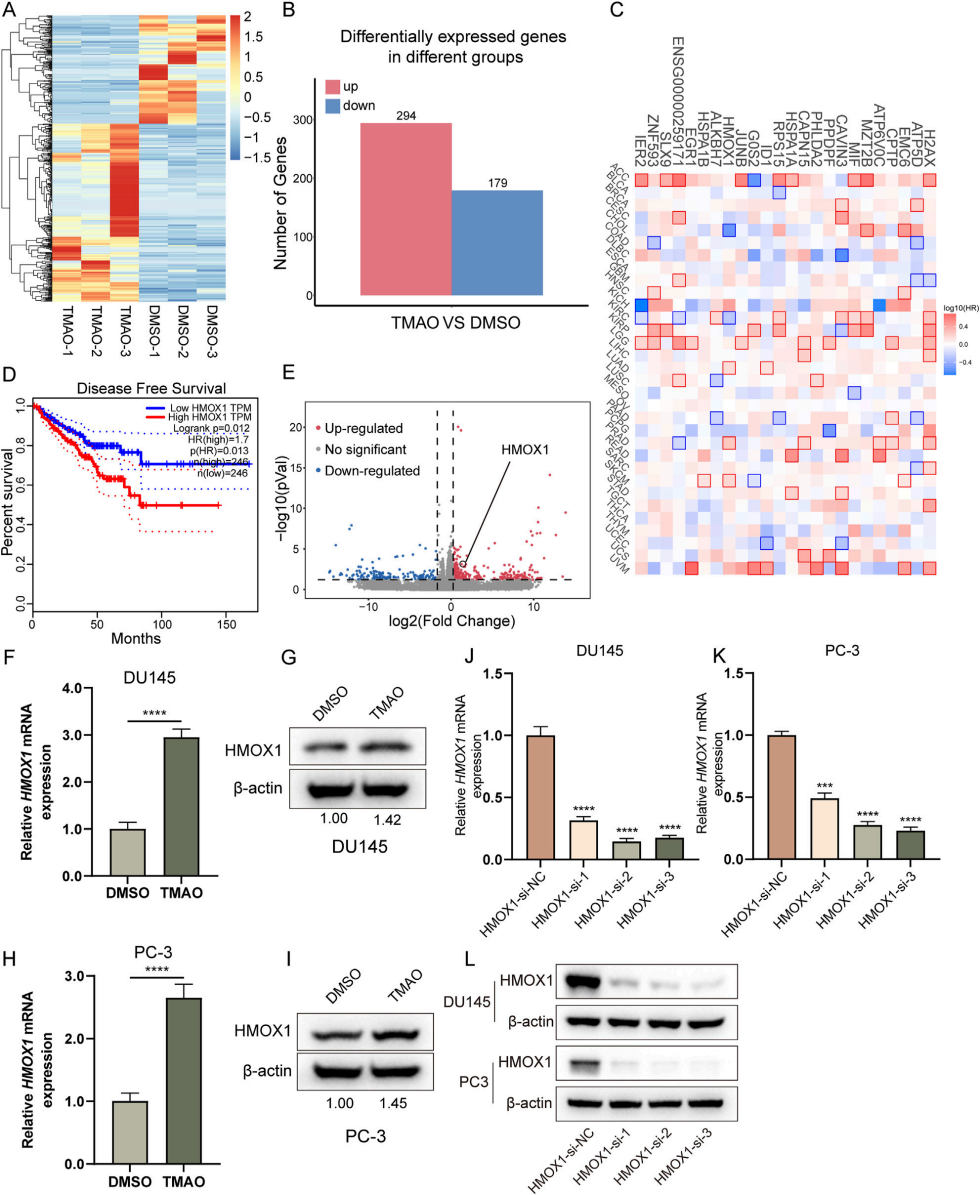
TMAO increases HMOX1 expression. **(A)** Overview of differentially expressed genes from RNA-seq; **(B)** Bar plot showing the number of upregulated and downregulated genes in PC3 cells after TMAO treatment; **(C)** Survival map displaying the prognostic significance of 24 selected genes across all cancers; **(D)** Kaplan-Meier curve showing the impact of HMOX1 expression on disease-free survival of patients; **(E)** Volcano plot highlighting the position of HMOX1 among differentially expressed genes; **(F–I)** Expression levels of HMOX1 at mRNA and protein levels in DU145 and PC3 cells after TMAO treatment; **(J–L)** qPCR and Western bloting analysis demonstrating the knockdown efficiency of HMOX1-siRNA.

To explore the mechanism by which TMAO promotes the proliferation and migration of prostate cancer cells, we performed RNA-seq analysis on PC3 cells treated with TMAO. The heatmap shows the overview of differentially expressed genes (Figure 2A). The barplot shows the number of upregulated and downregulated genes (Figure 2B). From these differentially expressed genes, we screened for statistically significant protein-coding genes with FPKM > 1 in each sample. Based on the above criteria, we obtained 24 genes, all of which are upregulated. These genes are listed in Supplementary Table 2. The survival map obtained from GEPIA2 showed the prognosis of patients in 33 cancers expressing these 24 genes (Tang et al., 2019), with red indicating poor prognosis for high expression and blue indicating good prognosis, with statistically significant differences marked by a frame (Figure 2C). The 24 genes were ranked by p-value, and we selected the top 10 genes. qPCR validation of these 10 genes revealed 7 stably differentially expressed genes, with heme oxygenase-1 (HMOX1) showing the greatest differential fold (Supplementary Figure S1C). Patients with high HMOX1 expression had worse prognosis (Figure 2D), and the volcano plot highlighted the *HMOX1* gene (Figure 2E). qPCR and Western blotting confirmed that TMAO treatment increased *HMOX1* mRNA and protein levels (Figures 2F–I). To investigate the impact of *HMOX1* on prostate cancer cells, we used siRNA to knock down *HMOX1*. qPCR and Western blotting validated the efficiency of HMOX1 knockdown (Figures 2J–L). *HMOX1*-si-2 and *HMOX1*-si-3 showed the best knockdown efficiency and were used for subsequent experiments.

### Knockdown of HMOX1 inhibits proliferation and migration of prostate cancer cells

**FIGURE 3:**
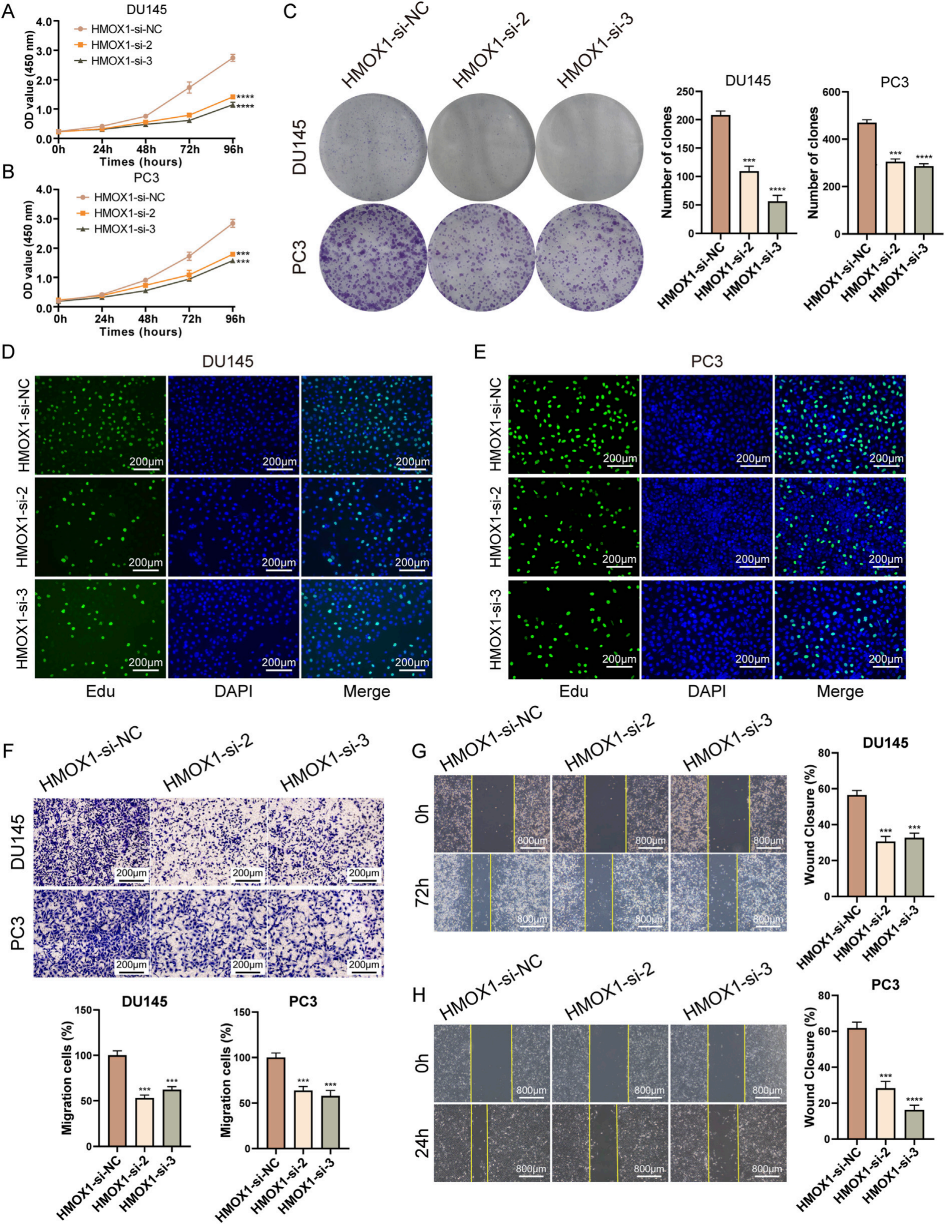
Knockdown of HMOX1 inhibits proliferation and migration of prostate cancer cells. **(A, B)** CCK8 assays showing the effect of HMOX1 knockdown on cell proliferation; **(C)** Colony formation assays demonstrating the impact of HMOX1 knockdown on cell proliferation capacity; **(D, E)** EdU assays showing the effect of HMOX1 knockdown on cell proliferation; **(F)** Transwell migration assays illustrating the effect of HMOX1 knockdown on cell migration; **(G, H)** Wound healing assays showing the impact of HMOX1 knockdown on cell migration.

In the CCK8 assay, we transfected prostate cancer cells with *HMOX1*-si-2 and *HMOX1*-si-3 for 96 h and found that *HMOX1* knockdown significantly inhibited cell proliferation (Figures 3A, B). Colony formation assay showed that *HMOX1* knockdown significantly reduced the number of colonies (Figure 3C). Edu assay confirmed that *HMOX1* knockdown significantly reduced the proportion of proliferating cells (Figures 3D, E). We also investigated whether *HMOX1* knockdown affects cell migration. Transwell assay showed that *HMOX1* knockdown significantly reduced the number of migrating cells (Figure 3F). The wound healing assay yielded similar results (Figures 3G, H). Overall, *HMOX1* acts as an oncogene promoting malignant progression in prostate cancer, and its knockdown significantly inhibits cell proliferation and migration.

### TMAO promotes proliferation and migration of prostate cancer cells through HMOX1

**FIGURE 4:**
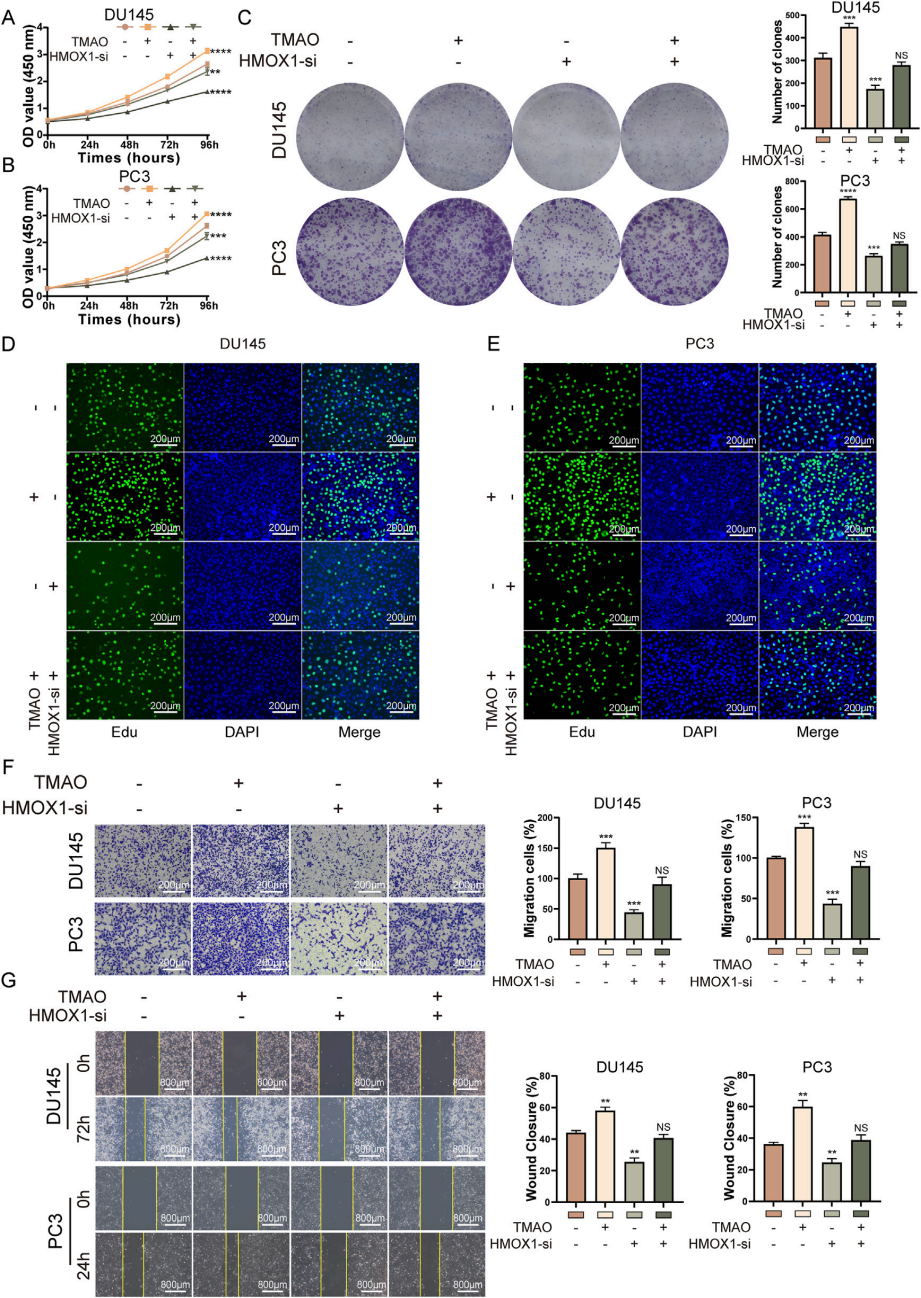
TMAO promotes proliferation and migration of prostate cancer cells through HMOX1. **(A, B)** CCK8 rescue assays indicate that HMOX1 knockdown can counteract TMAO-induced cell proliferation; **(C)** Colony formation assays show that HMOX1 knockdown reverses TMAO-enhanced cell proliferation; **(D, E)** EdU rescue assays demonstrate that HMOX1 knockdown can reverse TMAO-induced cell proliferation; **(F)** Transwell assays illustrate that HMOX1 knockdown can counteract TMAO-enhanced cell migration; **(G)** Wound healing assays show that HMOX1 knockdown can rescue TMAO-induced cell migration.

We conducted rescue experiments to determine if TMAO affects the proliferation and migration of prostate cancer cells through *HMOX1*. Given that *HMOX1*-si-3 showed better knockdown efficiency and greater impact on cell proliferation and migration, it was used for subsequent experiments. In the CCK8 assay, co-treatment with TMAO and *HMOX1*-si-3 showed that si-*HMOX1* counteracted the TMAO-induced proliferation of prostate cancer cells (Figures 4A, B). Colony formation and Edu assays produced similar results (Figures 4C–E). These experiments suggest that TMAO enhances cell proliferation through *HMOX1*. Transwell assay indicated that si-*HMOX1* reversed TMAO-induced migration of prostate cancer cells (Figure 4F). The wound healing assay yielded the same conclusion, as si-*HMOX1* counteracted TMAO-induced wound healing ability (Figure 4G). These experiments demonstrate that TMAO promotes the migration of prostate cancer cells through *HMOX1*.

### TMAO influences HMOX1 expression via p38 MAPK pathway

**FIGURE 5:**
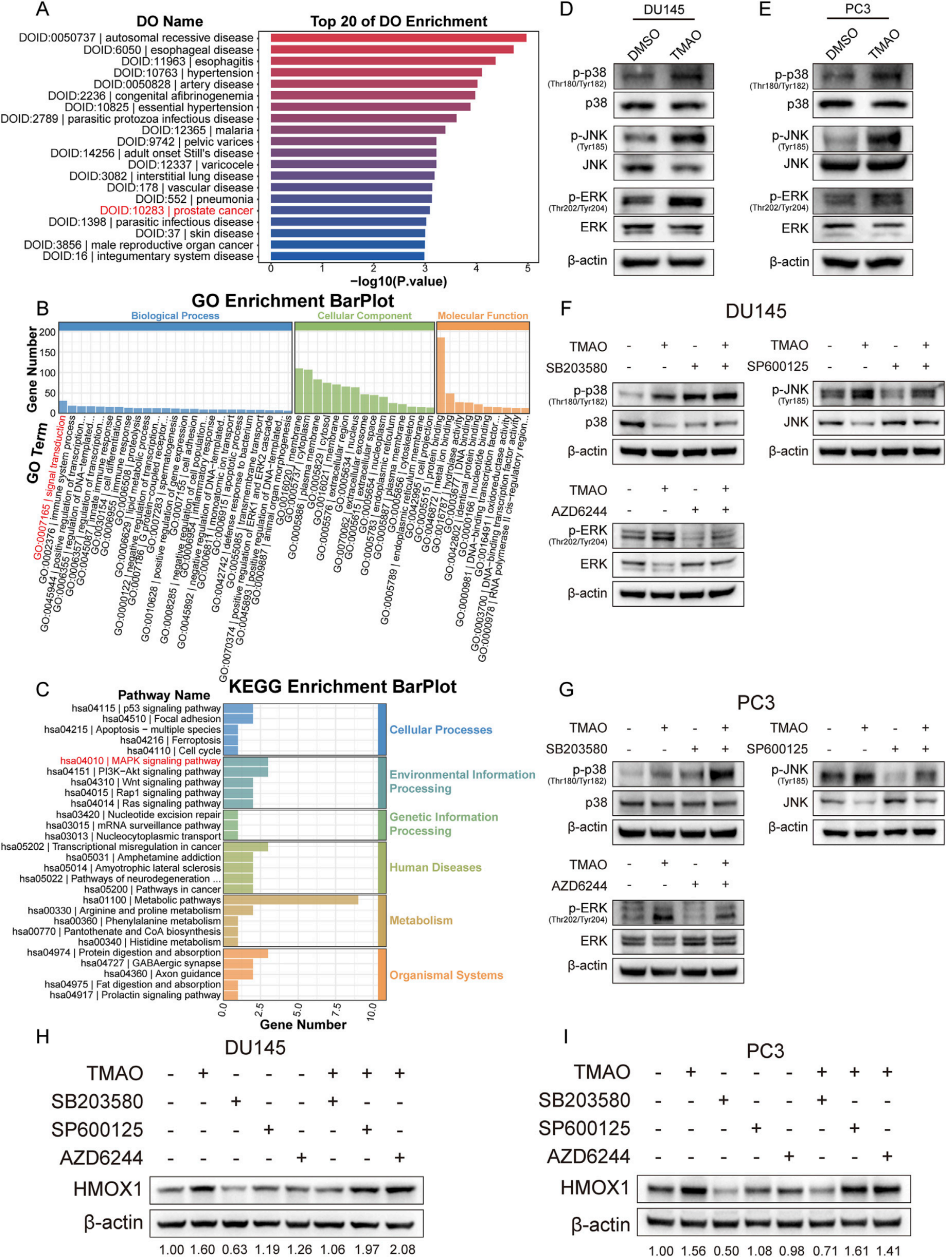
TMAO influences HMOX1 expression via p38 MAPK pathway. **(A)** Disease ontology analysis shows that genes altered by TMAO treatment are enriched in prostate cancer; **(B)** Gene ontology analysis reveals that TMAO treatment affects signal transduction; **(C)** KEGG pathway enrichment analysis indicates enrichment in the MAPK signaling pathway; **(D, E)** Western blotting shows activation of the MAPK signaling pathway in DU145 and PC3 cells after TMAO treatment; **(F, G)** Western blotting confirms the inhibitory efficiency of three small-molecule inhibitors: SB203580, SP600125, and AZD6244; **(H, I)** Western blotting demonstrates that TMAO increases HMOX1 expression through the p38 MAPK pathway.

Functional enrichment analysis of RNA-seq data from TMAO-treated cells showed enrichment of prostate cancer in disease ontology analysis (Figure 5A), supporting the hypothesis that TMAO affects prostate cancer progression. Gene ontology enrichment analysis indicated that TMAO treatment affects signal transduction process (Figure 5B). KEGG enrichment analysis revealed that TMAO might activate the MAPK signaling pathway (Figure 5C). The MAPK pathway plays a crucial role in cell differentiation, proliferation, invasion, metastasis, angiogenesis, and apoptosis, primarily involving extracellular signal-regulated kinase (ERK1/2), c-Jun N-terminal kinase (JNK), and p38 mitogen-activated protein kinase (p38 MAPK) (Zhang and Liu, 2002). Studies suggest that the p38 pathway in MAPK promotes HMOX1 expression (Lee et al., 2014). We hypothesized that TMAO activates HMOX1 expression through the MAPK pathway. Western blotting showed that TMAO activated the MAPK pathway in prostate cancer cells, increasing phosphorylated forms of p38, JNK, and ERK, while non-phosphorylated forms decreased or remained unchanged (Figures 5D, E). We used small molecule inhibitors SB203580, SP600125, and AZD6244 to inhibit the activation of p38, JNK, and ERK, respectively, and validated their efficiency (Figures 5F, G). Notably, SB203580 does not directly inhibit p38 phosphorylation but acts by inhibiting downstream pathways. In prostate cancer cells, TMAO treatment alone increased HMOX1 expression; SB203580 alone reduced HMOX1 expression. Co-treatment with TMAO and SB203580 counteracted the TMAO-induced HMOX1 expression increase (Figures 5H, I). Since HMOX1 is regulated by p38, inhibition of JNK and ERK did not reduce HMOX1 expression nor counteract TMAO-induced HMOX1 expression. In summary, TMAO promotes HMOX1 expression via the p38 MAPK pathway.

### High-choline diet promotes tumor growth and migration in mice

**FIGURE 6:**
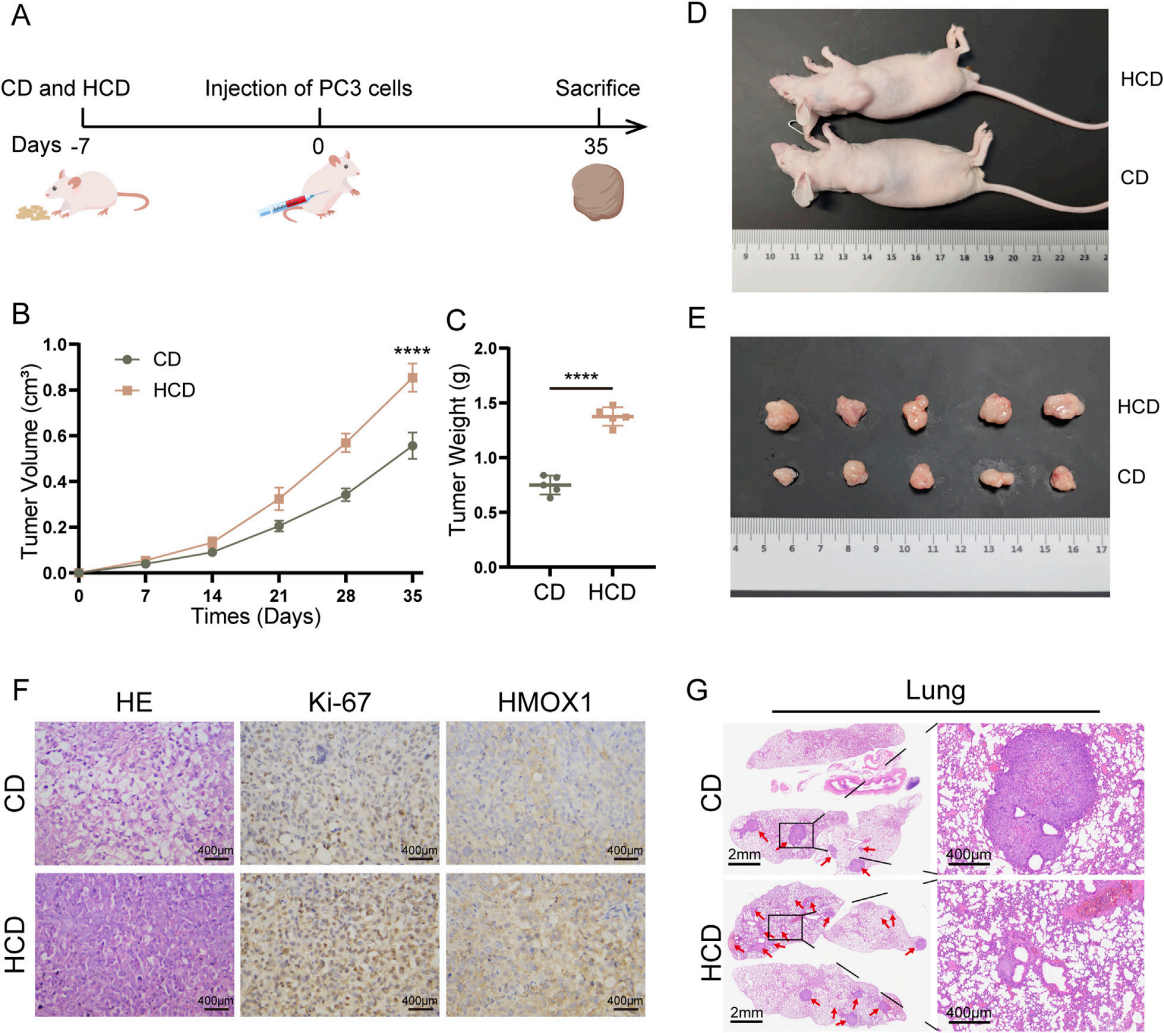
High-choline diet promotes subcutaneous tumor growth in mice. **(A)** Nude mice were fed a high-choline diet for 7 days prior to inoculation with PC3 cells on day 0. Subcutaneous tumor model mice remained on the diet until day 35, while lung metastasis model mice continued until day 60; **(B)** Tumor volumes in mice with control diet versus high-choline diet; **(C)** Comparison of tumor weights between the two groups after euthanasia; **(D)**, **(E)** Images of tumor sizes from both groups post-euthanasia; **(F)** HE staining and immunohistochemical staining of tumors from control diet and high-choline diet groups; **(G)** HE staining of lung metastasis tumor model.

Finally, we conducted *in vivo* experiments to verify the effect of TMAO. Mice were fed a control diet (CD) or a high-choline diet (HCD). After 7 days, PC3 cells were injected subcutaneously and via the tail vein in the mice. Subcutaneous tumor volume was measured every 7 days. Mice in the subcutaneous model group were euthanized on day 42, while mice in the lung metastasis model group were euthanized on day 67. Subcutaneous tumors and lung tissues were collected from mice. Tumor tissues were weighed and subjected to HE staining, Ki-67, and HMOX1 immunohistochemistry (Figure 6A). Results showed that tumors in HCD mice were significantly larger and heavier than those in CD mice (Figures 6B, C). Tumors were excised and displayed (Figures 6D, E). HE staining revealed that the cells in tumor samples from mice subjected to a high-choline diet intervention were more densely arranged. Immunohistochemistry revealed higher expression of Ki-67 and HMOX1 in tumors from HCD mice (Figure 6F). Additionally, the lung tissues of the mice were stained with HE. We found that mice in the HCD group had significantly more lung metastases than those in the CD group (Figure 6G). In conclusion, TMAO increases HMOX1 expression, promoting prostate cancer proliferation and migration.

## Discussion

PCa ranks as the fourth most commonly diagnosed cancer globally in 2022, and is the second leading cause of cancer incidence in men, with a significant impact on male cancer-related mortality (Bray et al., 2024). Despite high cure rates achievable with radical treatments, a substantial number of patients experience recurrence, leading to CRPC and poorer prognosis. Extensive research has identified various mechanisms driving prostate cancer progression, including androgen signaling, DNA repair systems, oncogenes and tumor suppressors, prostate-specific antigen, and transcription factors (Fontana et al., 2022). Additionally, factors such as innate and adaptive immunity, external environment, and gut microbiota have been implicated in influencing cancer development (Rizzo et al., 2022).

The gut microbiota plays a crucial role in maintaining human health and has been increasingly recognized for its role in cancer pathogenesis through various signaling mechanisms (Schwabe and Greten, 2020; Gilbert et al., 2018; Zmora et al., 2019). Recent research indicates that controllable factors such as obesity, exercise, smoking, and diet are involved in the progression of PCa (Boufaied et al., 2024). Short-term dietary changes directly impact the composition of the gut microbiota (David et al., 2014; Jumpertz et al., 2011). The study by Richman et al. reported a statistically significant positive correlation between a high-choline diet and the risk of fatal prostate cancer (Richman et al., 2011). Meanwhile, Keum et al. reported that a high-choline diet was mainly associated with fatal prostate cancer, but not with the overall risk of prostate cancer (Keum et al., 2015). Moreover, individual variations in gut microbiome composition, diversity, and abundance are significantly influenced by dietary habits and living environments (Zmora et al., 2019; Perler et al., 2023). Therefore, dietary interventions targeting the gut microbiota to modulate its composition and function could potentially delay or hinder the progression of prostate cancer, offering profound clinical implications. In this study, we demonstrated that the gut microbiota-derived metabolite TMAO influences the expression of HMOX1, thereby promoting proliferation and migration of prostate cancer cells. This finding provides a theoretical basis for understanding the relationship between gut microbiota and prostate cancer. It also explains how a high-choline diet may contribute to the risk of prostate cancer at the mechanistic level, providing a new perspective for future prevention and treatment, and highlighting the importance of dietary factors in cancer development.

When TMAO was first reported, it was considered a protein stabilizer capable of maintaining osmotic pressure in aquatic organisms and protecting proteins from urea, thermal denaturation, and stress-induced misfolding and refolding (Georgescauld et al., 2009). However, studies have yielded contrasting conclusions, revealing that TMAO can lead to protein instability and misfolding, thereby promoting cancer development (Rani et al., 2016). When the accumulation of misfolded or unfolded proteins reaches a certain level, it can activate the endoplasmic reticulum (ER) stress response. The PERK molecule, a component of the ER stress response located on the ER membrane, serves as a specific receptor for TMAO, indicating that TMAO can directly activate ER stress (Chen et al., 2019). Following sustained non-lethal ER stress, cancer cells adapt, grow, metastasize, and coordinate various immune escape mechanisms within the tumor microenvironment (TME), promoting malignant progression (Chen and Cubillos-Ruiz, 2021). From this perspective, TMAO’s promotion of proliferation and migration in colorectal cancer (Yue et al., 2017; Yang et al., 2022), liver cancer (Wu et al., 2022), and prostate cancer as studied here aligns with expectations. However, increasing evidence suggests that modulation of ER stress sensors or related factors can render invasive tumors significantly sensitive to cytotoxic drugs, targeted therapies, and immunotherapy (Chen and Cubillos-Ruiz, 2021). This aspect also explains findings by Wang et al. and Mirji et al., reporting TMAO as an “adverse” microbial metabolite that enhances tumor response to anti-PD-1 immunotherapy (Wang et al., 2022; Mirji et al., 2022). Thus, the reported effects of TMAO on tumors represent just the tip of the iceberg, with other mechanisms warranting further exploration.

HMOX1 is considered to exert cellular protective effects against oxidative stress by accelerating heme clearance, converting it into carbon monoxide, biliverdin, and iron (Tenhunen et al., 1968). Miller et al. reported that HMOX1 reduces ROS levels to protect prostate cancer cells from apoptosis and promotes progression to a castration-resistant phenotype (Miller et al., 2022). Moreover, it has been shown that exosomes produced by androgen-independent prostate cancer cells upregulate HMOX1, promoting the transition of androgen-dependent prostate cancer cells to androgen independence (Zhang et al., 2021). During this process, androgen-independent prostate cancer cells exhibit higher levels of HMOX1. Additionally, HMOX1 can undergo nuclear translocation via truncating a short C-terminal tail, acting as a transcription factor-like molecule that subsequently activates various downstream transcription factors, thereby initiating cascading signaling pathways (Lin et al., 2007). Furthermore, HMOX1 exerts extensive immune-regulatory roles within the TME, overall suppressing anti-tumor immune responses (Luu Hoang et al., 2021). A recent study has shown that HOMX1 on tumor-associated fibroblasts promotes iron accumulation in prostate cancer, leading to immunosuppression in prostate cancer. Targeted inhibition of HMOX1/iron/KDM6B signaling axis can reverse immunosuppressed TME (Zhang et al., 2024). This demonstrates the great therapeutic potential of targeting HMOX1.

In tumors, multiple factors contribute to increased HMOX1 expression. Firstly, tumor cells experience constant oxidative stress, leading to elevated ROS levels (Vafa et al., 2002). Secondly, hemoglobin levels are known to increase in cancer, promoting HMOX1 expression in a substrate-dependent manner (Fukuda et al., 2017; Fiorito et al., 2019). Additionally, hypoxia, a common feature of tumors and their TME, stabilizes hypoxia-inducible factor-1α (HIF-1α), which in turn initiates *HMOX1* gene expression (Lee et al., 1997; Berra et al., 2001). Lastly, the *HMOX1* promoter region contains multiple transcription factor binding sites, allowing regulation by various cytokines to modulate its gene expression (Luu Hoang et al., 2021). In summary, there are diverse pathways through which HMOX1 expression can be elevated, and increased HMOX1 levels contribute to cancer progression through both tumor cell intrinsic mechanisms and modulation of the TME. Therefore, further understanding the role of the antioxidant HMOX1 in prostate cancer or other cancers is crucial. Moreover, it may serve as a therapeutic target or biomarker for preventing or monitoring the transition from hormone-sensitive prostate cancer to hormone-resistant forms.

This study has limitations. Firstly, our focus on TMAO as the primary metabolite influenced by a high-choline diet precluded metabolomics analysis to identify other significant changes. Additionally, we did not explore changes in gut microbiota structure and abundance following a high-choline diet in mice.

## Conclusion

In conclusion, our study provides novel evidence of the role of gut microbiota-derived metabolite TMAO in prostate cancer. We found that TMAO activates the p38 pathway to enhance HMOX1 expression, thereby promoting proliferation and migration of prostate cancer cells. These findings suggest that reducing choline intake through dietary interventions may delay the onset and progression of prostate cancer, offering significant clinical implications. Furthermore, HMOX1 emerges as a potential target or biomarker for monitoring and potentially slowing the transition of prostate cancer to CRPC. Further research into these mechanisms could pave the way for new therapeutic strategies and personalized interventions in prostate cancer management.

## Data Availability

The datasets presented in this study can be found in the NCBI Sequence Read Archive (SRA) under the BioProject ID PRJNA1206539. The data can be accessed at the following link: https://www.ncbi.nlm.nih.gov/bioproject/PRJNA1206539.

## Abbreviations

PCa, Prostate cancer; CRPC, Castration-resistant prostate cancer; TMA, Trimethylamine; TMAO, Trimethylamine-N-oxide; ROS, Reactive oxygen species; FBS, Fetal bovine serum; HE, Hematoxylin and eosin; HMOX1, Heme oxygenase-1; ERK1/2, Extracellular signal-regulated kinase; JNK, c-Jun N-terminal kinase; MAPK, Mitogen-activated protein kinase; ER, Endoplasmic reticulum; TME, Tumor microenvironment; HIF-1α, Hypoxia-inducible factor-1α; CD, Control diet; HCD, High-choline diet.
