# Supplementary material for: Gut microbiota derived metabolite trimethylamine N-oxide influences prostate cancer progression via the p38/HMOX1 pathway
Source: Front Pharmacol. 2025 Jan 9;15:1526051. doi: 10.3389/fphar.2024.1526051 (PMC11754881; doi:10.3389/fphar.2024.1526051)
Supplement: Supplementary file 4 [file DataSheet1.docx]

**Gut Microbiota Derived Metabolite Trimethylamine N-Oxide Influences Prostate Cancer Progression via the p38/HMOX1 Pathway**

**Supplementary Materials**


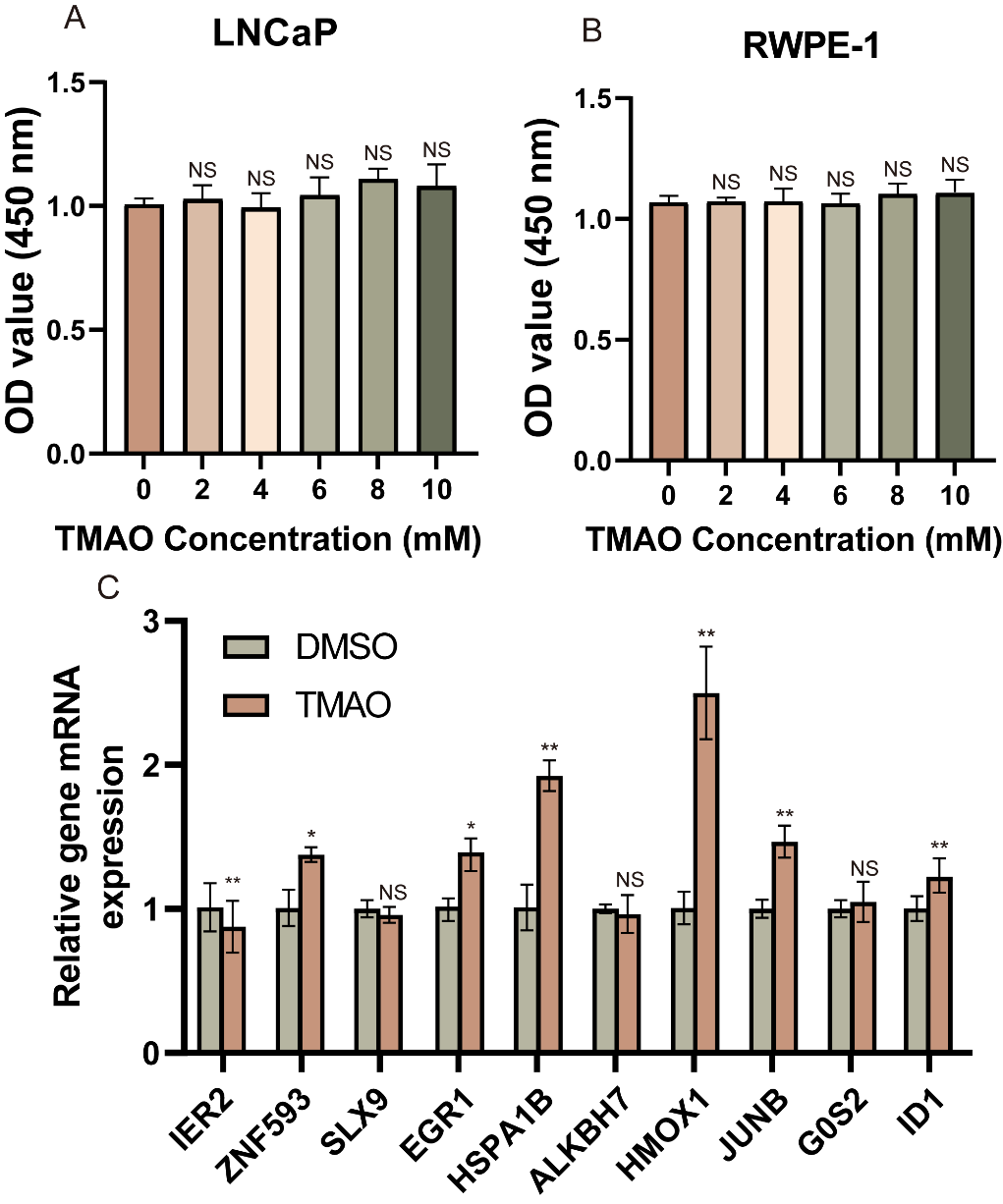
The supplementary materials included one supplementary figure and three supplementary tables.

**Supplementary Figure 1.** (**A**) CCK8 assay showing LNCaP cells treated with different concentrations of TMAO; (**B**) CCK8 assay showing REPE-1 cells treated with different concentrations of TMAO; (**C**) qPCR analysis of the expression of 10 selected genes in PC3 cells after TMAO treatment. * means p value < 0.05; ** means p value < 0.01.

**Supplementary Table 1.** Primers and HMOX1-siRNA sequences used in this experiment.

**Supplementary Table 2.** RNA-seq data of the 10 selected differentially expressed genes.

**Supplementary Table 3.** Raw differentially expressed genes from the RNA-seq data.
